# The end of a dogma: the safety of doxycycline use in young children for malaria treatment

**DOI:** 10.1186/s12936-017-1797-9

**Published:** 2017-04-13

**Authors:** Tiphaine Gaillard, Sébastien Briolant, Marylin Madamet, Bruno Pradines

**Affiliations:** 1 Fédération des Laboratoires, Hôpital d’Instruction des Armées Desgenettes, Lyon, France; 2 Unité de Parasitologie et d’Entomologie, Département des Maladies Infectieuses, Institut de Recherche Biomédicale des Armées, HIA Laveran, Boulevard Laveran, 13013 Marseille, France; 3 Aix Marseille Université, Unité de Recherche sur les Maladies Infectieuses et Tropicales Emergentes, UM 63, CNRS 7278, IRD 198, Inserm 1095, Institut Hospitalo-universitaire Méditerranée Infection, Marseille, France; 4 Centre National de Référence du Paludisme, Marseille, France

**Keywords:** Antibiotics, Doxycycline, Malaria, *Plasmodium falciparum*, Anti-malarial drug, Resistance, Prophylaxis, Treatment, Children

## Abstract

Anti-malarial drug resistance to chloroquine and sulfadoxine–pyrimethamine has spread from Southeast Asia to Africa. Furthermore, the recent emergence of resistance to artemisinin-based combination therapy (ACT) in Southeast Asia highlights the need to identify new anti-malarial drugs. Doxycycline is recommended for malaria chemoprophylaxis for travel in endemic areas, or in combination with the use of quinine for malaria treatment when ACT is unavailable or when the treatment of severe malaria with artesunate fails. However, doxycycline is not used in young children under 8 years of age due to its contraindication due to the risk of yellow tooth discolouration and dental enamel hypoplasia. Doxycycline was developed after tetracycline and was labelled with the same side-effects as the earlier tetracyclines. However, recent studies report little or no effects of doxycycline on tooth staining or dental enamel hypoplasia in children under 8 years of age. In the United States, the Centers for Disease Control and Prevention have recommended the use of doxycycline for the treatment of acute and chronic Q fever and tick-borne rickettsial diseases in young children. It is time to rehabilitate doxycycline and to recommend it for malaria treatment in children under 8 years of age.

## Background

According to the latest World Health Organization (WHO) estimates, nearly half of the world’s population lived in malaria endemic areas in 2015 [1]. The burden of malaria predominantly affects sub-Saharan Africa accounting for 90% of the 212 millions of malaria cases and 90% of the 429,000 deaths. Most of the cases of malaria in 2015 were in Africa (90%). Children are especially vulnerable and represented more than 90% of the malaria cases in Africa. The highest proportion of deaths was averted in Africa (94%). Of the estimated 6.8 million fewer malaria deaths between 2001 and 2015, about 6.6 million (97%) were for children aged under 5 years. In 2015, 303,000 malaria deaths (range: 165,000–450,000) were estimated to have occurred in children aged under 5 years, which is equivalent to 70% of the global total. Malaria remains a major killer of children, taking the life of a child every 2 min. The spread of anti-malarial drug resistance from Southeast Asia to Africa has previously occurred with chloroquine and sulfadoxine–pyrimethamine [2, 3]. The emergence of resistance to artemisinins, manifested by delayed parasite clearance after monotherapy with artesunate or ACT, was described in Southeast Asia [4, 5]. Drug resistance is currently emerging even to the most recently commercialized ACT, dihydroartemisinin–piperaquine, in Cambodia, where 84% of treated patients showed delayed parasite clearance half-lives in 2012–2014 [6–8]. Antibiotics, and more specifically doxycycline, have been shown to be effective against malaria parasites [9–11]. Doxycycline is recommended for malaria chemoprophylaxis for travel in endemic areas [12, 13]. Considering doxycycline delayed onset of action, this finding justifies its therapeutic use in combination with a fast schizonticide [11]. The addition of doxycycline for severe malaria was much more as an attempt to reduce the emergence of resistance, and reduce the use of artesunate or quinine monotherapy. Doxycycline can alter recrudescent parasites. Additionally, doxycycline can also prevent bacterial infections which could be associated with malaria. Doxycycline is recommended in combination with quinine for malaria treatment in the event of ACT unavailability in some European clinical practice [14, 15]. This combination is also used when treatment of severe malaria with artesunate fails [16]. In some African hospitals, oral doxycycline is used systematically in combination with intravenous artesunate in the treatment of severe *P. falciparum* malaria [17]. Additionally, some severe *P. vivax* cerebral malaria cases have been treated with artesunate and doxycycline [18, 19]. However, doxycycline is contraindicated in children under 8 years of age because of the risk of yellow tooth discolouration and dental enamel hypoplasia.

In this review, the potential effects of doxycycline on teeth were re-analysed. Information sources were identified using PubMed, Google Scholar and Google. Searches were performed in these electronic databases using the search term ‘doxycycline’ alone or in combination with ‘teeth’ or ‘children’ or ‘side-effects’.

## Potential for adverse dental effects

Tetracycline compounds have been used since 1953. Doxycycline was developed in 1967. Unfortunately, doxycycline was labelled with the same side-effects as had been reported for the tetracyclines, including the risk of yellow tooth discolouration and dental enamel hypoplasia in children under 8 years of age.

In the early 1960s, dental side-effects were associated with the use of tetracycline. Children suffering with cystic fibrosis who were treated with tetracycline (41–63 children) presented a high prevalence (38–80%) of discoloured teeth after long-term treatment [20–22]. The incidence of permanent staining of teeth was greater with high doses of tetracycline and long-term administration, but tetracycline also pigmented the teeth of 23–92% of babies or children after short courses of treatment (55/238, 16/23 and 46/50 babies) or led to hypoplasia of the enamel [23–25]. In a large study in 520 children, tetracycline was determined to be the probable causative agent of dental defects in 112 cases (21.5%) [26]. Due to these results, all the tetracyclines were contraindicated in children under 8 years of age because of the risk of yellow tooth discolouration and dental enamel hypoplasia. However, it was shown in experimental animals that tooth discolouration was more associated with tetracycline than with chlortetracycline or oxytetracycline [27]. Doxycycline has a lower affinity to chelate calcium (19%) compared to tetracycline (39.5–74.5%), leading to a reduced risk of yellow tooth discolouration and dental enamel hypoplasia [28–31]. Studies using doxycycline in children have reported little or no effect on teeth. Lochary and co-workers did not find any significant difference in dental staining between 10 children treated with doxycycline and 20 control children without treatment [32]. A randomized, blind, controlled study in young children with refractive atypical pneumonia did not detect tooth staining in either group (31 children with doxycycline and 30 control non-doxycycline exposed children) [33]. Most of the children were under 4 years of age when they were treated with doxycycline. Doxycycline was administered in syrup form at a dose of 4 mg/kg twice a day on the first day followed by a single dose of 2 mg/kg for nine days. Some children received up to four courses of doxycycline before the age of 8 years (mean of 2.0 courses per child). In another study, 58 children who lived in an American–Indian reservation were treated with doxycycline for suspected Rocky Mountain spotted fever (RMSF) with a mean duration of 7.3 days before the age of eight [34]. These children received multiple courses of doxycycline, with a mean of 1.8 courses per child and a total of 107 courses for the group. There was no difference in yellow tooth discolouration and dental enamel hypoplasia between children who were treated with doxycycline and 213 non-exposed children.

## Discussion

These different studies showed that short courses of doxycycline in children under 8 years of age had no visible dental effects, such as staining of teeth or enamel hypoplasia. Recently, discolouration of teeth was observed (2.8%) in adults receiving prolonged courses of doxycycline (100 mg twice a day for 6 months) for abdominal aortic aneurysms [35].

Doxycycline is a cheap drug with a broad therapeutic spectrum and very little evidence of serious adverse events [36]. It is time to recommend the use of doxycycline in children under 8 years of age for short course treatments [37–40]. Since 2013, the Centers for Disease Control and Prevention (CDC) have recommended the use of doxycycline at a dose of 2.2 mg/kg twice per day for two weeks in children under 8 years of age for the treatment of acute and chronic Q fever in United States [41]. The CDC Rickettsial Zoonoses Branch recommended the use of doxycycline for treatment of tick-borne rickettsial diseases in the United States in 2016 [42]. It is time now to use doxycycline in young children for malaria treatment in combination with artesunate or quinine, in line with its recommended use for adults and children older than 8 years of age [14–16]. For chemoprophylaxis for young children travellers (long-term course), the recommendations should remain unchanged [12, 13].

Additionally, intermittent preventive treatment in infants (IPTi) is recommended by the WHO [43]. Intermittent preventive treatment in children (IPTc), especially in schoolchildren (IPTsc), is a proven malaria control strategy [44–46]. In Africa, school-aged children represent 26% of the population, and nearly half of these children have at least one malaria infection each year [47]. In evaluating different schedules, children were treated every 5 months, 4 months, 3 months, 2 months or every month with sulfadoxine–pyrimethamine, sulfadoxine–pyrimethamine–amodiaquine, sulfadoxine–pyrimethamine–artesunate, amodiaquine–artesunate, or dihydroartemisinin–piperaquine [46, 48–52]. Doxycycline, which is a low-half-life drug (20 h) [11], could be associated with long-half-life drugs such as amodiaquine, piperaquine or sulfadoxine–pyrimethamine in young children for IPTsc. An advantage of using doxycycline is its mechanism of action which differs from those of most currently used anti-malarial drugs by inhibiting proteins implicated in apicoplast metabolism [11, 53, 54].

However, the use of doxycycline for malaria chemoprophylaxis could raise the risk of selecting resistant bacterial pathogens to antibiotics [55]. The microbiome plays an important role in human health, and changes in the microbial flora can lead to resistance or infection by pathogenic bacteria. Proliferation of pathogenic bacteria in the human microbiome has been documented following antibiotic exposure [56, 57]. Q fever endocarditis patients treated with doxycycline presented significantly lower concentrations of *Bacteroidetes*, *Firmicutes* and *Lactobacillus* [58], a finding that was also observed with the anti-malarial hydroxychloroquine [58]. Anti-malarial drugs certainly have an impact on the microbiota. It is important to determine the effects of doxycycline on the microbiome, especially if multiple antibacterial combinations are used like sulfadoxine–pyrimethamine and doxycycline, especially in young children. The impact of long-term chemoprophylaxis with doxycycline on bacterial pathogens is documented only in military clinical cases with the acquisition of multi-drug resistance by bacteria [59–62]. However, these increases in multi-drug-resistant bacteria were likely associated with environmental exposures rather than doxycycline exposure. Only outbreaks of Panton-Valentine leukocidin-positive, doxycycline-resistant, methicillin-susceptible *Staphylococcus aureus* infections have been linked to long-term chemoprophylaxis with doxycycline in soldiers under deployment [63]. There is no published information on the development of bacterial resistance after short-courses of doxycycline used in malaria prophylaxis.

## Conclusions

Doxycycline was developed after tetracycline and was labelled with the same side-effects as the earlier tetracyclines, including the risk of yellow tooth discolouration and dental enamel hypoplasia in children under 8 years of age. However, recent studies report little or no effects of doxycycline on tooth staining or dental enamel hypoplasia in children under 8 years of age. In the United States, the CDC have recommended the use of doxycycline for the treatment of acute and chronic Q fever and tick-borne rickettsial diseases in young children. It is time to rehabilitate doxycycline and to recommend it in combination with the use of quinine for malaria treatment when ACT is unavailable or when the treatment of severe malaria with artesunate fails, in children under 8 years of age. Doxycycline could also be associated with long-half-life drugs such as amodiaquine, piperaquine or sulfadoxine–pyrimethamine in young children for IPTsc.

## Abbreviations

ACT: artemisinin-based combination therapy
CDC: Centers for Disease Control and Prevention
IPTc: intermittent preventive treatment for children
IPTi: intermittent preventive treatment for infants
IPTsc: intermittent preventive treatment for schoolchildren
WHO: World Health Organization
